# Supplementary material for: Post-traumatic stress symptoms are associated with better performance on a delayed match-to-position task
Source: PeerJ. 2018 May 3;6:e4701. doi: 10.7717/peerj.4701 (PMC5936632; doi:10.7717/peerj.4701)
Supplement: Supplemental Information 1 [file peerj-06-4701-s001.docx]

**DETAILS OF DDM ANALYSIS**

The EZ2 package version 1.2.r11 ([Grasman, 2015](#_ENREF_5)) was installed from <http://R-Forge.R-project.org> and run under R version 3.3.3 "Another Canoe" ([R Core Team, 2017](#_ENREF_9)) on a Macintosh MacBook running macOS Sierra (10.12.5) with 2.4 GHz Intel Core 2 Duo processor.

The input file ("./dotsforEZ2.csv") was a comma-delimited file in the current working directory, containing a header row ("cVRT27", "cVRT63", "Pe27", "Pe63", "cMRT27", "cMRT63") followed by 118 rows each containing summary data for one subject, where cMRT is mean RT for correct trials (in sec), cVRT is variance of RT on correct trials (in sec^2^), and Pe is proportion of error trials (i.e. trials with incorrect responses) at the Low (27 pixels) and Medium (63 pixel) spatial separation.

The below code was then executed to load the EZ2 package, read in the datafile, assign starting parameter values based loosely on prior published studies, define the model, and execute the model to estimate parameters for each subject:

library(EZ2)

myData <- read.table("./dotsforEZ2.csv", header=TRUE, sep=",")

startValues1 <- c(v27=0.1, v63=0.2, z=0.05, a=0.9, Ter=0.2)

mdl1 <- list(cVRT27~EZ2.cvrt(v27,z,a),

cVRT63~EZ2.cvrt(v63,z,a),

Pe27~EZ2.pe(v27,z,a),

Pe63~EZ2.pe(v63,z,a),

cMRT27~EZ2.cmrt(v27,z,a,Ter),

cMRT63~EZ2.cmrt(v63,z,a,Ter))

EZ2batch(startValues1, mdl1, data=myData, nrestart=1)

The batch job (fitting the model to all 118 subjects) required 98.78 s (i.e. <1 s per subject) as reported by the system.time() function (with no particular attempt to suppress other ongoing processor usage while the batch job was in progress).

The resulting group means (v27=0.235, v63=0.228, z=0.680, a=1.480, Ter=0.201) were then used as starting values for another EZ2 batch run; since about a third of simulations failed to converge after a single pass, nrestart was set to 20 to ensure as many simulations converged as possible (system time required approx. 17 min.). The batch job was repeated to verify that identical values were produced on multiple runs.
